# ﻿Two new species of *Entoloma* subg. *Cyanula* sect. *Asprella* (Entolomataceae, Agaricales) from subtropical regions of China

**DOI:** 10.3897/mycokeys.126.173472

**Published:** 2025-12-02

**Authors:** Yu-Qin Xu, Hui Zeng, Liu-Hua Guo, Jun-Qing Yan, Sheng-Nan Wang

**Affiliations:** 1 Jiangxi Provincial Key Laboratory of Excavation and Utilization of Agricultural Microorganisms, Jiangxi Agricultural University, Nanchang 330045, China Jiangxi Agricultural University Nanchang China; 2 Institute of Edible mushroom, Fujian Academy of Agricultural Sciences, Fuzhou 350011, China Institute of Edible mushroom, Fujian Academy of Agricultural Sciences Fuzhou China; 3 Jiangxi Provincial Key Laboratory of Subtropical Forest Resource Cultivation, College of Forestry, Jiangxi Agricultural University, Nanchang 330045, China iangxi Agricultural University Nanchang China

**Keywords:** Basidiomycetes, new taxa, phylogeny, taxonomy

## Abstract

Two new species of Entoloma subg. Cyanula, *E. qingluan* and *E. zilin*, are described from subtropical regions of China based on morphological and phylogenetic evidence. *E. qingluan* is characterized by blue basidiomata, paler greyish-blue lamellae with a poliopus-type lamella edge, and pileipellis cells containing dark brown vacuolar pigments. *E. zilin* features a greyish-magenta scaly pileus with a bluish lamella edge adjacent to the stipe, a blue stipe, fertile lamella edges, and predominantly 2-spored basidia. Both species are assigned to subg. Cyanula sect. Asprella based on their striate or squamulose pileus, blue stipe, and fertile or poliopus-type lamella edge. Detailed descriptions, color photos, and scanning electron micrographs of spores are presented.

## ﻿Introduction

*Entoloma* (Fr.) P. Kumm. is a species-rich and morphologically diverse genus in the Agaricales Underw. (Kirk et al. 2008) and the largest genus in the Entolomataceae Kotl. & Pouzar. It is widely distributed across five continents, having been found in regions ranging from frigid Arctic zones to temperate areas and tropical regions. Most species of *Entoloma* are saprotrophic fungi, commonly thriving in shady and damp forest zones, on litter, as well as on soil and decaying wood. Additionally, some species are capable of parasitizing other fungi or plants (Horak 1980; Noordeloos 1981; Ediriweera et al. 2017). More than 2,000 species within this genus have been reported worldwide (Romagnesi 1941; Romagnesi and Gilles 1979; Horak 1980, 1982; Largent 1994; Horak 2008; Noordeloos and Gates 2012). The primary characteristics of this genus include spore prints ranging in color from pink, angular basidiospores observed from all angles, and remarkable morphological diversity, including mycenoid, collybioid, omphalioid, clitocyboid, and pleurotoid forms (Co-David et al. 2009; Baroni and Matheny 2011).

Historically, the taxonomic position of species within Entoloma subg. Cyanula has been debated. Romagnesi (1974) first treated them as a section within *Rhodophyllus* Quél. sensu lato. Later, they were transferred to Entoloma subg. Leptonia, which was divided into three sections: sect. Leptonia, sect. Cyanula, and sect. Griseorubida (Noordeloos 2004; Noordeloos and Gates 2012). However, subsequent molecular phylogenetic studies revealed that subg. Leptonia is polyphyletic. Specifically, sect. Leptonia belongs to the /*Nolanea*–Claudopus clade, whereas sect. Cyanula and sect. Griseorubida fall within the /*Inocephalus*–*Cyanula* clade (Co-David et al. 2009; Noordeloos and Gates 2012; Largent et al. 2016). Morphologically, species within sect. Leptonia exhibit clamp connections, whereas those in sect. Cyanula lack such structures. Based on molecular and morphological evidence, sect. Cyanula was elevated to the rank of subgenus (Noordeloos and Gates 2012; Reschke et al. 2022a). In the monograph of Noordeloos et al. (2022a), the authors reclassified species with a striate or squamulose pileus, blue stipe, and either fertile lamellae edge or sterile, poliopus-type lamella edge into sect. Asprella of subg. Cyanula. The section can be divided into six subsections, namely, subsect. Asprella, subsect. Exilia, subsect. Cistocruentata, subsect. Cyaneoliacina, subsect. Rivipollensia, and subsect. Perfidodisca.

Taxonomic studies on species within subg. Cyanula remain limited in China. To date, only 17 taxa within subg. Cyanula have been documented in China, including four species newly described in recent studies (Chen et al. 2025). Over the past few years, during our investigations into macrofungal diversity in subtropical regions of China, we have found that species within subg. Cyanula exhibit remarkably high diversity, with numerous potentially undescribed taxa. This study describes two novel species within subg. Cyanula sect. Asprella, based on comparative morphological characterization and phylogenetic analyses.

## ﻿Materials and methods

### ﻿Morphological studies

The specimens in this study were collected from Fujian and Zhejiang Provinces in China between 2022 and 2023 and were preserved as dried specimens. All specimens are deposited in the Herbarium of Fungi, Jiangxi Agricultural University (HFJAU). Fresh specimens were photographed in the field and recorded macroscopically. Color notations adhered to the Methuen Handbook of Colour (Kornerup and Wanscher 1978). Micromorphological structures were observed and measured under an Olympus BX53 microscope (Olympus Corporation, Tokyo, Japan) by making squash preparations of sections of dried specimens that were placed in 5% KOH solution or H_2_O. A 1% Congo red solution was used as the staining agent for observing colorless tissues. Amyloidity was tested with Melzer’s reagent (Horak 2005; Chen et al. 2024). For each collection, the dimensions of at least 40 basidiospores, basidia, and cystidia were measured. The size range of spores is expressed in the format (a) b–c (d), where “a” and “d” represent the minimum and maximum values, respectively, and 90% of the spores fall within the range of “b–c.” The meanings of other spore characteristics are as follows: “Q” represents the length-to-width ratio; “av” indicates the average value; “n” denotes the number of measurements; and “Qm” represents the average “Q” value ± standard deviation (Yu et al. 2020). Morphological descriptions are based on the work of Noordeloos et al. (2022a). The morphology of the spores was further verified using an electron microscope. A portion of the gills from the dried fruiting body was sampled and observed under an electron microscope (JEOL JSM-IT800 Schottky Field Emission Scanning Electron Microscope).

### ﻿DNA extraction, PCR amplification, and sequencing

Genomic DNA was extracted from dried specimens using the NuClean Plant Genomic DNA kit (CWBIO, China) (Wang et al. 2022). The nrDNA ITS and LSU regions were amplified using the primer pairs ITS1F/ITS4 and LR0R/LR5 or LR7 (White et al. 1990; Jr and Vilgalys 1999).

PCR amplification was conducted using a 25 µL reaction system as follows: 1 µL of DNA, 1 µL of each forward and reverse primer, 9.5 µL of ddH_2_O, and 12.5 µL of 2× TaqMaster Mix [Qing Ke Biotechnology Co. Ltd. (Wuhan City, China)]. PCR was performed using a touchdown program for all regions: initial 95 °C for 5 min; 14 cycles of denaturing at 95 °C for 30 s, annealing at 65 °C for 45 s (with a decrease of 1 °C per cycle), and extension at 72 °C for 1 min; followed by 30 cycles of denaturing at 95 °C for 30 s, annealing at 52 °C for 30 s, and extension at 72 °C for 1 min; and a final extension at 72 °C for 10 min (Bau and Yan 2021). The PCR products were sequenced by Qing Ke Biotechnology Co. Ltd. (Wuhan City, China).

### ﻿Alignment and phylogenetic analyses

A total of 89 sequences (62 ITS sequences and 27 LSU sequences) from 62 samples were used for phylogenetic analyses using Bayesian inference (BI) and maximum likelihood (ML). Sequence selection was based on the results of BLAST for ITS and the study of Noordeloos et al. (2022a). Species of E. subg. Cubospora served as outgroups (Table 1). ITS and LSU sequences were aligned separately with the MAFFT online server using the automatic selection of algorithm (Katoh et al. 2019). Concatenated sequences were analyzed with MRBAYES v.3.2.7a (Ronquist et al. 2012) and IQ-TREE v.2.1.2 (Nguyen et al. 2015), respectively. For the ML analysis, models of sequence evolution were assessed in IQ-TREE prior to analysis, allowing partitions of the sequences to have different seeds (-spp). The selected models were TPM2u+F+I+G4 for ITS and TPM3u+F+I+G4 for LSU. Ultrafast bootstrap support values were calculated from 1,000 replicates. For the BI analysis, the Markov chain Monte Carlo runs were set for 2 million generations. The first 25% of trees were discarded as burn-in. Nodes with Bayesian posterior probabilities (BI-PP) ≥ 0.95 and ML ultrafast bootstrap proportions (ML-BP) ≥ 95% were considered statistically supported (Nguyen et al. 2015).

**Table 1.** Details of sequences used in the phylogenetic analyses. Newly generated sequences were in bold.

Species	Location	Voucher Number	ITS	LSU	Sequence origin
*Entoloma argus*	Vietnam	LE F-312694 holotype	OM987263	OM996175	(Morozova et al. 2022)
*E. arion*	Vietnam	LE F-312691 holotype	OM987259	OM996176	(Morozova et al. 2022)
*E. asprellum*	Estonia	TUF106064	UDB011486	—	UNITE
*E. azureosquamulosum*	China	GDGM27355 holotype	NR_137086	NG_059214	(He et al. 2012)
*E. caespitosum*	China	GDGM24025	JQ281490	JQ410327	(He et al. 2012)
*E. caespitosum*	China	GDGM24026	JQ281491	JQ320133	(He et al. 2012)
*E. calceus*	Norway	O-F-259457 holotype	NR_182489	—	(Noordeloos et al. 2022b)
*E. calceus*	France	LIP0402265	ON008492	—	(Noordeloos et al. 2022b)
*E. callipygmaeum*	Russia	LE253784 holotype	MZ145207	—	(Dima et al. 2021)
*E. carneogriseum*	Norway	O-F-256479	UDB07673714	—	UNITE
*E. chalybeum*	Russia	LE254353	KC898445	KC898500	(Morozova et al. 2014)
*E. chalybeum*	Denmark	TUF105760	UDB034191	—	UNITE
*E. chloropolium*	Estonia	TUF120516	UDB031513	—	UNITE
*E. cistocruentatum*	Spain	L 0607521	NR_182485	—	(Noordeloos et al. 2022b)
*E. coracis*	Norway	O-F-256850 holotype	MW934571	MW934251	(Crous et al. 2021b)
*E. corvinum*	France	FA4261	OR419868	—	(Armada et al. 2023)
*E. cyaneolilacinum*	Norway	O-F-252009 holotype	MW934582	MW934252	(Crous et al. 2021b)
*E. cyanostipitum*	China	GDGM31318 holotype	KY711237	KY972694	(He et al. 2017)
*E. cycneum*	Vietnam	LE F-343654 holotype	OQ779461	OQ804518	(Morozova and Pham 2023)
*E. dislocatum*	Spain	L0607565 holotype	ON008483	—	(Noordeloos et al. 2022b)
*E. exile*	Germany	Lueck8	KP965773	KP965791	(Karich et al. 2015)
*E. exile*	USA	K(M)187354	MF977976	—	Unpublished in GenBank
*E. griseocyaneum*	Russia	LE254351	KC898444	KC898498	(Morozova et al. 2014)
*E. griseocyaneum*	Germany	KaiR997	MZ611684	—	(Reschke et al. 2022b)
*E. icarus*	Vietnam	LE F-312696 holotype	OM987257	OM996174	(Morozova et al. 2022)
*E. incanum*	Sweden	LE312503 neotype	OK161247	OK161275	(Crous et al. 2021b)
*E. incanum*	Russia	LE311794	OK161249	OK161276	(Crous et al. 2021b)
*E. isborscanum*	Russia	LE302088 holotype	MW934566	MW934253	(Crous et al. 2021a)
*E. kovalenkoi*	Vietnam	LE312529 holotype	OK257210	OK257207	(He et al. 2014)
*E. linkii*	Norway	O-F-256353	UDB07673651	—	UNITE
*E. mastoideum*	China	GDGM28820	JQ281476	JQ410328	(He et al. 2012)
*E. meridionale*	Greece	ACAM2018-0153 holotype	OL679700	—	(Dima et al. 2022)
*E. mougeotii*	Estonia	TUF101633	UDB016265	—	UNITE
*E. mougeotii*	Estonia	TUF106505	UDB019720	—	UNITE
*E. murrayi*	China	QI 1001	KJ658967	JQ993090	(He et al. 2014)
*E. mutabilipes*	Finland	TUR610/12	LN850550	—	(Kokkonen 2015)
*E. notabile*	Cyprus	L-0607514 holotype	OL343537	—	(Vila et al. 2021)
*E. pallidostriatum*	Spain	L-0607566 holotype	NR_177630	—	(Vila et al. 2021)
*E. perasprellum*	France	GC01100310 holotype	MZ145177	—	(Dima et al. 2021)
*E. perchalybeum*	Sweden	GB-0209474 holotype	NR_182490	—	(Noordeloos et al. 2022b)
*E. perfidodiscum*	Spain	L-0607586 holotype	NR_177633	—	(Vila et al. 2021)
*E. phlebophyllum*	China	HFJAU3126	OR827451	OR826040	(Chen et al. 2024)
*E. poliopus*	Estonia	TUF120264	UDB024655	—	UNITE
*E. pseudosubcorvinum*	Thailand	SDBR-CMUNK0985 holotype	MZ215769	MZ203540	(Bhunjun et al. 2022)
*E. pulchripes*	Russia	LE311808 holotype	MZ145188	—	(Dima et al. 2021)
*E. pulchripes*	Russia	LE311809	MZ145189	—	(Dima et al. 2021)
***E. qingluan***	**China**	**HFJAU5122 holotype**	**PX426788**	**PX426795**	**This work**
***E. qingluan***	**China**	**HFJAU5723**	**PX426789**	**PX426796**	**This work**
*E. queletii*	Turkey	OKA-TR1002	MT741747	—	Unpublished in GenBank
*E. queletii*	Estonia	TUF141044	UDB07674927	—	UNITE
*E. riparium*	Italy	L-0607563 holotype	NR_177632	—	(Vila et al. 2021)
*E. rivipollense*	Spain	L-0607585 holotype	NR_177634	—	(Vila et al. 2021)
*E. septentrionale*	Norway	O-F-254295 holotype	NR_174647	—	(Noordeloos et al. 2021)
*E. serrulatum*	Russia	LE254361	KC898447	KC898501	(Morozova et al. 2014)
*E. serrulatum*	Iran	EnSe-1	KT833862	—	Unpublished in GenBank
*E. sicoense*	Portugal	PO-F2244 holotype	OR026624	—	(Fachada et al. 2023)
*E. sicoense*	Portugal	PO-F2245	OR026625	—	(Fachada et al. 2023)
*E. subcaesiocinctum*	China	SAAS133 holotype	KY711236	KY972697	(He et al. 2017)
*E. subtenuicystidiatum*	China	GDGM 28459 holotype	JQ320109	JQ320116	(He et al. 2012)
*E. timidum*	Russia	LE 312480 holotype	MZ145197	—	(Dima et al. 2021)
*E. turci*	Austria	WU25055	UDB0802163	—	UNITE
***E. zilin***	**China**	**HFJAU3354 holotype**	**PX426790**	**PX426797**	**This work**

## ﻿Results

### ﻿Phylogenetic analysis

A total of 1,715 characters were used in the phylogenetic analyses (ITS, 827 bp; LSU, 888 bp), of which 991 were constant, 554 were parsimony-informative, and 170 were singleton. For the Bayesian analysis, the average standard deviation of split frequencies was less than 0.01 after 1,625,000 generations.

**Figure 1.**
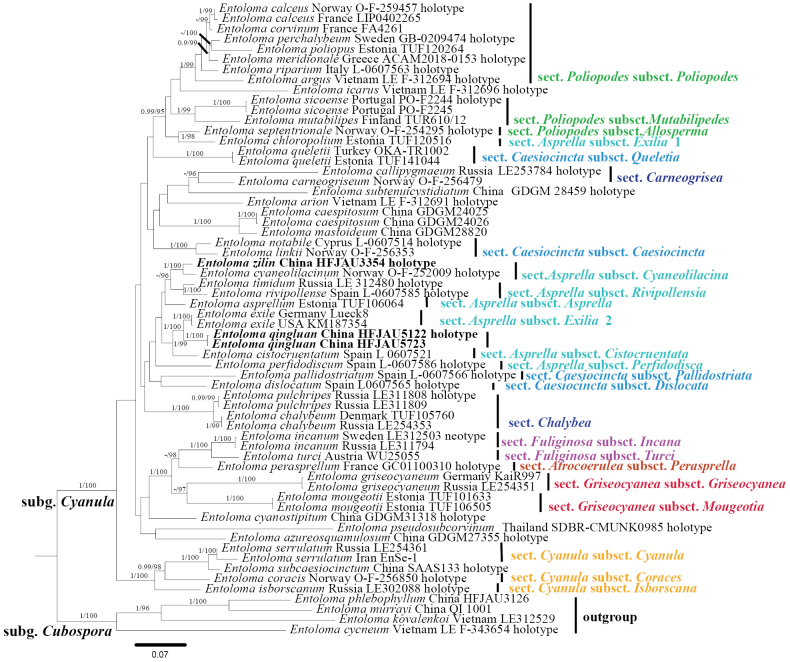
Phylogram of Entoloma subg. Cyanula spp. generated by Bayesian inference (BI) analysis based on ITS and LSU, rooted with E. subg. Cubospora spp. Bayesian inference (BI-PP) ≥ 0.95 and ML ultrafast bootstrap proportions (ML-BP) ≥ 95% are indicated as PP/BP. The new taxa are marked in bold.

The result of the phylogenetic analysis is shown in Fig. 1. The two new species were clustered in subg. Cyanula sect. Asprella and formed separate and well-supported branches. *E. qingluan* formed a separate and well-supported lineage (BI-PP = 1, ML-BP = 100%) and grouped with *E. exile* (Fr.) Hesler. *E. zilin* belongs to /sect. Asprella subsect. Cyaneolilacina and is closely related to *E. cyaneolilacinum*.

### ﻿Taxonomy

#### ﻿ Entoloma qingluan

Taxon classificationFungiAgaricalesEntolomataceae

J.Q. Yan, Y.Q. Xu & S.N. Wang sp. nov.

B5AC2507-D11D-5C05-9637-AB662D58A161

MycoBank No: 860910

Fig. 2

##### Etymology.

Derived from Hanyu Pinyin, the epithet alludes to the Qingluan bird of the *Classic of Mountains and Seas*, whose plumage matches the fungus’s color.

**Figure 2.**
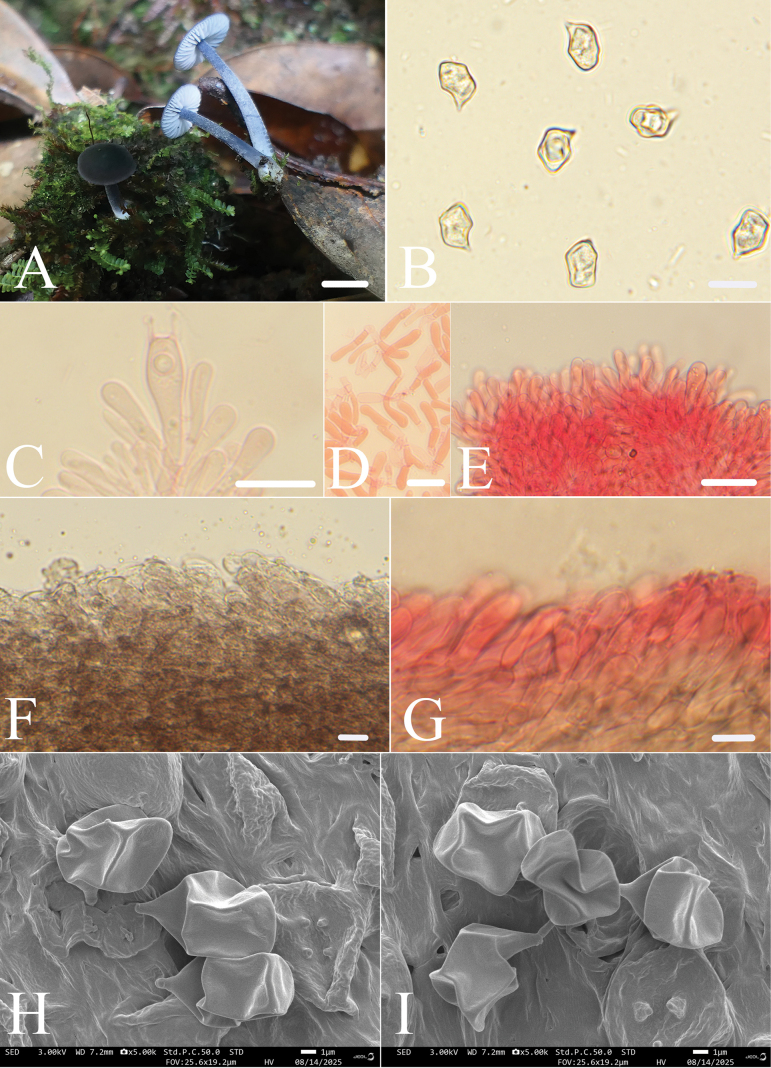
*Entoloma qingluan*. **A.** Basidiomata; **B, H, I.** Basidiospores; **C.** Basidia; **D, E.** Cheilocystidia; **F, G.** Pileipellis. All microscopic structures were observed in 5% KOH, and 1% Congo red was used as the stain except **F**. Scale bars: 5 mm (**A**); 10 μm (**B**); 20 μm (**C–E**); 10 μm (**F, G**).

##### Chinese name.

青鸾粉褶菌.

##### Holotype.

China • Fujian Province, Wuyishan, 16 August 2023, collected by Nian-kai Zeng, Cheng-feng Nie, Hua-zhi Qin, Hui Deng, Tian Jiang, Run-xiang Zhao, HFJAU5122.

##### Diagnosis.

*Entoloma qingluan* is mainly characterized by the rather small, tricholomatoid basidiomata; a convex, bluish grey to blackish blue pileus; paler greyish blue lamellae with sterile lamellae edge; narrow clavate cheilocystidia, and pileipellis cells containing dark brown vacuolar pigments; clamp connections absent.

##### Macromorphology.

Basidiomata rather small, tricholomatoid. Pileus 6.0–8.0 mm wide, convex, slightly umbonate, bluish grey to blackish blue (20F3–5), darker at center, paler toward margin, margin entire, slightly involute. Lamellae moderately distant, 0.5–1.5 mm wide, adnate, ventricose, with two tiers of lamellulae, paler greyish blue (21C5), edge serrate to crenate, concolorous. Stipe 7.0–16.5 × 1.2–2.0 mm, central, terete, slightly broadened downward, hollow, blue-black to dark blue or greyish blue (20E5–20D4), the surface is covered with white, velutinous to fibrillose-scales, base with white tomentum. Odor and taste not recorded.

##### Micromorphology.

Basidiospores (7.5)8.5–10.5 × 6.5–8.0(8.5) μm, av = 9.4 × 7.2 μm, Q = 1.2–1.3 (Qm = 1.29 ± 0.03, n = 52), heterodiametrical, 5–7-angled in profile view, thick-walled, appearing nodulose, inamyloid. Basidia 37.0–54.5 × 9.0–12.5 μm, clavate, 4-spored. Pleurocystidia absent. Lamellae edge sterile, poliopus-type. Cheilocystidia clustered on lamella edge, 9.5–25.5 × 3.0–6.0 μm, narrowly clavate to subcylindrical. Lamellar trama regular, hyphae cells 3.5–6.0 µm wide. Pileipellis a trichoderm made up of clavate terminal cells, 5.5–16.0 μm, slightly constricted or level at the septa, with rounded or acute ends and dark brown vacuolar pigment. Stipitipellis transitional between cutis and trichoderm, composed of hyphae 4.0–8.0 μm wide, slightly constricted at septa, apices rounded, with brilliant granules abundant. Clamp connections absent.

##### Habitat.

Scattered on mixed forest floors dominated by broad-leaved trees, such as *Pinus* spp. and *Pterocarya* spp.

##### Additional specimens examined.

China • Fujian Province, Wuyishan, 16 August 2023, collected by Nian-kai Zeng, Cheng-feng Nie, Hua-zhi Qin, Hui Deng, Tian Jiang, Run-xiang Zhao, HFJAU5723.

##### Notes.

Considering the characteristics of this species, such as a darker-colored center on the pileus, blue stipe, and sterile lamella edges of the poliopus-type, this species can be classified into the subg. Cyanula sect. Asprella (Noordeloos et al. 2022a). However, it is impossible to classify *E. qingluan* into any of the subsections under this group based solely on morphological characteristics. Morphologically, this species is most similar to *E. cistocruentatum* Vila, Noordel. & Dima. However, *E. cistocruentatum* has fertile lamellae edges, lacks cheilocystidia, and possesses blue pigment in the pileipellis (Noordeloos et al. 2022b).

Morphologically, the new species is easily confused with *E. cyaneolilacinum* Noordel., J.B. Jordal, Brandrud & Dima. Both have a blue pileus and stipe, similar-sized spores, and brown vacuolar pigment in the pileipellis. However, the latter has a pileus with translucent stripes, emarginate lamellae that are white with a faint bluish tinge, fertile edges, and lacks cheilocystidia (Crous et al. 2021a). In addition, *E. azureosquamulosum* Xiao L. He & T.H. Li, discovered in southern China, also shows considerable similarity to this species. Both exhibit tricholomatoid basidiomata, with a blue pileus, adnate lamellae, sterile lamella edges, regular lamellar trama, and trichoderm pileipellis. However, *E. azureosquamulosum* has white to pink lamellae with a blue-tinged edge, fusoid cheilocystidia, and caulocystidia (He et al. 2012).

#### ﻿ Entoloma zilin

Taxon classificationFungiAgaricalesEntolomataceae

J.Q. Yan, Y.Q. Xu & S.N. Wang sp. nov.

4BC75BCA-73C1-56DF-A950-543E2A7025F4

MycoBank No: 860912

Fig. 3

##### Etymology.

The name “zilin” is derived from Hanyu Pinyin. The dark purple color of this species reminds people of the mythical zilin in Chinese legends, which is also depicted as dark purple.

**Figure 3.**
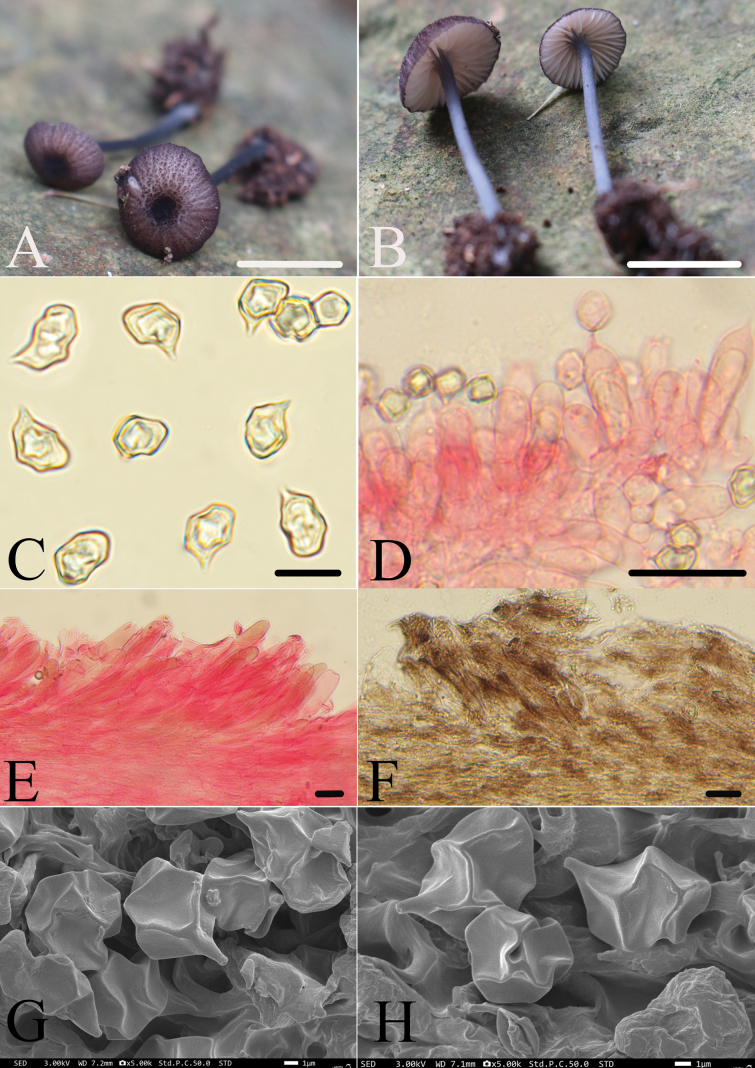
*Entoloma zilin*. **A, B.** Basidiomata; **C, G, H.** Basidiospores; **D.** Fertile lamella edge; **E, F.** Pileipellis. All microscopic structures were observed in 5% KOH, and 1% Congo red was used as the stain except **F**. Scale bars: 10 mm (**A–C**); 30 μm (**D**); 20 μm (**E, F**).

##### Chinese name.

紫麟粉褶菌.

##### Holotype.

China • Zhejiang Province, Wenzhou City, Wencheng County, Ya-ping Hu, 25 May 2022, HFJAU3354.

##### Diagnosis.

*Entoloma zilin* is mainly characterized by the rather small, collybioid basidiomata; pileus surface colored greyish-magenta which is covered with fibrous scales and is also striate; lamellae decurrent to slight sinuate, white with a bluish edge adjacent to the stipe; stipe blue; lamella edge fertile; cheilocystidia absent; pileipellis cells with sepia brown vacuolar pigment; clamp connections absent.

##### Macromorphology.

Basidiomata small, collybioid. Pileus 8.0–10.0 mm wide, convex with slight central umbilicate depression, not hygrophanous, margin entire, surface densely covered with fibrous scales, greyish-magenta (14E6), margin and center dark purple (14F5), striate from the margin to the center. Lamellae moderately distant, decurrent to slight sinuate, white with a slight pinkish tinge, edge entire, bluish marginate adjacent to the stipe, with two tiers of lamellulae. Stipe 20.0–25.0 × 1.0–1.5 mm, central, terete, equal, hollow, dark blue (20E5); surface covered with white velutinous scales; base with white mycelium. Odor and taste not recorded.

##### Micromorphology.

Basidiospores (8.5)9.0–12.0(13.0) × (6.5)7.0–8.5 μm, av = 10.7 × 7.6 μm, Q = 1.2–1.6(1.7) (Qm = 1.4 ± 0.1, n = 50), heterodiametrical, 5–8-angled in profile view, appearing nodulose, thick-walled, inamyloid. Basidia 29.0–41.0 × 7.0–12.0 μm, clavate, mainly 2-spored, rarely 4-spored. Lamellae edge fertile. Cheilocystidia and pleurocystidia absent. Lamellar trama regular. Pileipellis a trichoderm of cells 7.0–19.0 μm in diameter, cylindrical, slightly constricted at the septa, with cylindrical these terminal cells filled with sepia brown vacuolar pigment. Stipitipellis a cutis of densely arranged cylindrical hyphae 7.0–19.0 μm wide, slightly constricted at the septa, with terminal cells cylindrical to narrowly clavate and with rounded apices. Clamp connections absent.

##### Habitat.

Scattered on the ground surface of broad-leaved forests and attached to the soil.

##### Notes.

Based on the presence of scales and striations on the pileus surface, a blue stipe, and a fertile lamellae edge, the new species can be classified into sect. Asprella subsect. Cyaneolilacina. Only one species is known in this subsection: *E. cyaneolilacinum* Noordel., J.B. Jordal, Brandrud & Dima. However, it can be clearly distinguished from the new species by its deep blue to paler lilac-blue pileus, up to 25 mm in diam., and the absence of 2-spored basidia (Crous et al. 2021a).

The new species is morphologically similar to *E. violaceoviride* Arnolds & Noordel. Both have a greyish-magenta pileus with striations and scales. However, the latter has a papillate umbo on the pileus, adnexed lamellae that are initially violaceous then become violaceous-pink, and a stipe that is pale greyish green at first, and then gradually changes from the base upward to bluish-grey with a greenish tinge, with the apex remaining pale green, as well as sterile lamellae edges (Noordeloos 2004). In addition, the new species also shares certain similarities with *E. melongenicolor* Noordel. & Hauskn. from Africa (Mahé Island, Seychelles). Both have umbilicate pileus centers, blue stipes, similarly sized spores, fertile lamellae edges, and cheilocystidia absent. However, the latter has an aubergine-colored pileus, pale brick-red lamellae, and a pileipellis with bluish-brown vacuolar pigment (Noordeloos and Hausknecht 2016).

## ﻿Discussion

In this study, the two newly discovered species, *E. qingluan* and *E. zilin*, each form an independent and well-supported branch in the phylogenetic tree based on ITS and LSU sequences and are therefore considered genetically distinct from their closest relatives. Morphologically, both new species can be classified as sect. Asprella. Nevertheless, sect. Asprella is not recovered as a monophyletic clade: *E. zilin* nests within sect. Asprella subsect. Cyaneolilacina alongside *E. cyaneolilacinum*, whereas *E. qingluan* clusters with *E. exile* (sect. Exilia). The morphological differences between *E. zilin* and *E. cyaneolilacinum* have been discussed in the notes. *Entoloma qingluan* is easily separated from *E. exile* by its blue-grey to blue-black pileus and stipe; *E. exile* has a yellow-brown or grey-brown pileus, often with olivaceous or whitish tints, and its stipe turns red when bruised.

At the sectional level, subgenus Cyanula displays extensive incongruence between morphology and molecular phylogeny. Section Asprella is resolved as polyphyletic, and the same applies to sections *Poliopodes* and *Caesiocincta*. In addition, numerous taxa cannot yet be assigned to defined sections or subsections. To refine intrasectional delimitation, additional loci, broader sampling, and continued discovery of new taxa are imperative. Only by integrating molecular and morphological data can the current polyphyletic structure of subgenus Cyanula be resolved. The present study enhances our understanding of the diversity of this subgenus in China.

## Supplementary Material

XML Treatment for Entoloma qingluan

XML Treatment for Entoloma zilin

## ﻿Additional information

### Use of AI

No use of AI was reported.

### Author ORCIDs

Yu-Qin Xu https://orcid.org/0009-0002-0735-8323

Hui Zeng https://orcid.org/0000-0003-2025-844X

Jun-Qing Yan https://orcid.org/0000-0003-1128-5171

Sheng-Nan Wang https://orcid.org/0000-0003-0648-271X

### Data availability

All of the data that support the findings of this study are available in the main text.
